# Implementation of a tele-paediatric network in hospitals in a rural region: A mixed methods implementation study

**DOI:** 10.1177/20552076251350924

**Published:** 2025-07-20

**Authors:** Nils Pfeuffer, Angelika Beyer, Luisa Tischler, Sarah Heimbuch, Heiko Krause, Markus Krohn, Steffen Fleßa, Thomas Ruppel, Wolfgang Hoffmann, Neeltje van den Berg

**Affiliations:** 1 Section Epidemiology of Health Care and Community Health, Institute for Community Medicine, 60634 University Medicine Greifswald, Greifswald, Germany; 2 partner site Greifswald/Rostock, German Center for Child and Adolescent Health (DZKJ), Greifswald, Germany; 3 Institute for Nursing Science and Interprofessional Learning, 60634 University Medicine Greifswald, Greifswald, Germany; 4 Chair of General Business Administration and Health Care Management, University of Greifswald, Greifswald, Germany; 5 Rechtsanwaltsgesellschaft Dr. Ruppel mbH, Lübeck, Germany

**Keywords:** Telemedicine, videoconferencing, paediatrics, rural health services, health services accessibility

## Abstract

**Background:**

In rural states, paediatric capacities and competencies are concentrated in urban centres due to demographic change and economical pressure. This results in greater distances, longer waiting times and multiple examinations for rural-dwelling children seeking healthcare. A regional tele-paediatric network (RTP-Net) was implemented to improve the access of children to paediatric care by providing videoconferencing and digital health information exchange between paediatricians from hospitals with different sizes and degrees of specialization.

**Objective:**

Evaluation of the user acceptance as well as implementation barriers and facilitators.

**Methods:**

Between January 2021 and March 2024, mixed-methods were carried out including participant observation and a survey of the RTP-Net users. Descriptive analysis based on the Technology Acceptance Model according to Davis. The study report follows ‘A comprehenSive tool to Support rEporting and critical appraiSal of qualitative, quantitative and mixed methods implementation reSearch outcomes’ (ASSESS).

**Results:**

A total of 507 tele-consultations were conducted. 907 questionnaires were analysed. The respondents were satisfied with 92.1% of the tele-consultations. The respondents found most of the tele-consultations (95.8%) adequate for solving their questions. Technical issues were the most mentioned reason (59.3%) why tele-consultation were inadequate; 184 project diary entries were analysed; 42 times (61%), the participants highlighted positive usability aspects of the RTP-Net (e.g., improved availability of patient health data) and 27 times (39%) negative aspects (e.g., interoperability issues).

**Conclusions:**

A regional tele-paediatric healthcare network is feasible and acceptable in order to improve paediatric care in rural regions. However, technical improvements and policy adjustments are needed to enhance telemedicine adoption.

Trial registration: This study was registered under the title ‘Implementation und Evaluation of a Regional Tele-paediatric Network in Mecklenburg-Vorpommern and Brandenburg' (registration no. DRKS00024002, https://drks.de/search/de/trial/DRKS00024002) at the German Registry for Clinical Trials.

## Plain language summary

Development of a Virtual Healthcare Network for Children in Hospitals in a Rural Region: Technology Acceptance Study.

Due to low birth rates and high-cost pressure, hospital departments for children care in rural regions sometimes have to close. Children's doctors with certain medical specializations are also rare in rural regions. Therefore, children living in rural regions often have a limited access to specialized healthcare. A regional network providing videoconferencing and clinical data exchange was implemented in a rural region in order to connect hospitals of different sizes and different skills with each other. Children living in rural regions can benefit from such a hospital cooperation, as it offers shorter waiting times for children and a specialist's opinion is available more quickly. However, doctors’ uptake of new communication and information technologies is low in Germany. Therefore, the aim of this study was to find out which aspects affect the doctors’ use of a virtual care network.

We used a combination of different types of methods in order to gain an in-depth understanding of the doctors’ motivations for participation.

Between January 2021 and March 2024, the network physicians conducted 507 virtual consultations. We collected 184 project diary entries and received 907 questionnaires; 42 times (61%), the participants highlighted positive aspects of the virtual network (e.g., improved availability of children health data, improved children's access to healthcare); 27 times (39%), the participants mentioned negative aspects (e.g., technical issues). The overall user satisfaction was very high (92%). Technical issues were often caused by bad internet signal or local firewall settings.

A regional telemedical healthcare network for children is useful for improving children's access to healthcare care and is highly accepted by the participating doctors. However, due to tight working schedules of the doctors and the scattered German healthcare landscape, the provided technologies need to be as much accessible and easy to use as possible.

## Introduction

### Context

Due to demographic ageing on the one hand and the labour-intensive delivery of paediatric healthcare on the other hand, labour shortages in the healthcare system can be observed in most of the high- and middle-income countries and will increase by 2030.^1–3^ Additionally, inpatient paediatric healthcare in Germany stands under high-cost pressure, since the remuneration of paediatric healthcare services by the German Diagnosis Related Groups (G-DRG) system is insufficient.^4^ Especially in rural regions with greater distances between patients and paediatric healthcare providers a centralization of paediatric specialties or even a lack of certain specialities can be seen.^5,6^ Under these circumstances, coverage with high-quality and patient-centred healthcare services for rural-dwelling children is jeopardized.

### Solution

Telemedicine, usually defined as the use of modern information and communication technologies for providing healthcare services over greater distances,^7^ can support paediatric healthcare in underserved regions.^8^ For example, remote clinical consultancy and assessments as well as the remote management of acute care children living in rural areas can avoid unnecessary transportations and associated costs^9–11^ and helps to detect health issues of rural-dwelling children earlier. Telemedical consultations to assess urgency of medical interventions by paediatricians have been shown to be highly reliable in comparison with face-to-face consultations.^5^ Video-assisted consultations can increase the physicians’ confidence when assessing paediatric patients’ health from remote.^5,12^ Moreover, telemedicine can bring specialized paediatric services to hospitals where the availability of paediatricians and/or sub-specialized paediatricians is limited^13^ and, also, advancing mHealth technologies enable specialists to remotely monitor children’s health.^14,15^

However, there are also barriers to the implementation of telemedicine, like currently unaccounted additional workload for implementing and practicing the use of telemedicine, administrative burden and lack of digital technology access.^16^ Moreover, technical barriers can cause a low uptake of telemedicine by the users.^17^

Mecklenburg Western Pomerania, in the Northeast of Germany, is a rural federal state facing many of the above-mentioned challenges of paediatric care for rural-dwelling children. That is why, a regional tele-paediatric care network (RTP-Net) has been implemented in this region in order to connect quaternary/tertiary care hospitals with secondary care hospitals and increase the access of rural-dwelling children to specialized paediatric care. The implementation followed a Community-Based Participatory Research (CBPR)^18^ approach by including the perspectives of the addressed users and stakeholders of the RTP-Net in all phases of the project beginning with conceptualizing the telemedicine up to implementing and disseminating the RTP-Net system in the participating hospitals.

### Research questions

The following publication pursues the main research question:

1. How effectively was the RTP-Net system implemented, as measured by the users’ technology acceptance and satisfaction?

Secondary research questions are:

2. What aspects of the RTP-Net system act as facilitators or barriers to its implementation and use?
3. What improvements to the RTP-Net system are needed to enhance users’ technology acceptance and engagement?

## Methods and materials

### Study design

The design of the study is a prospective mixed-methods implementation study. The RTP-Net study was conducted between April 2020 and March 2024. The study's information and results are reported in this publication according to ‘A comprehensive tool to Support reporting and critical appraisal of qualitative, quantitative and mixed-methods implementation research outcomes’ (ASSESS).^19^ We used the checklist of the ASSESS tool to systematically address all aspects relevant to the reporting of the study details. The mixed-methods approach was employed in order to use the qualitative results for informing the interpretation of the quantitative results as well as to analyse qualitative data with descriptive statistics. The benefit of this approach is that it contextualizes the quantitative findings which allow interpretations of the reasons why users were or were not satisfied with the provided RTP-Net system in certain cases and which adjustments of the system and/or the policies are needed. In addition, the use of mixed-methods allows for the quantification of qualitative data and provides insights into the importance of certain previously identified themes for the study population.

### Setting

**Figure 1.**
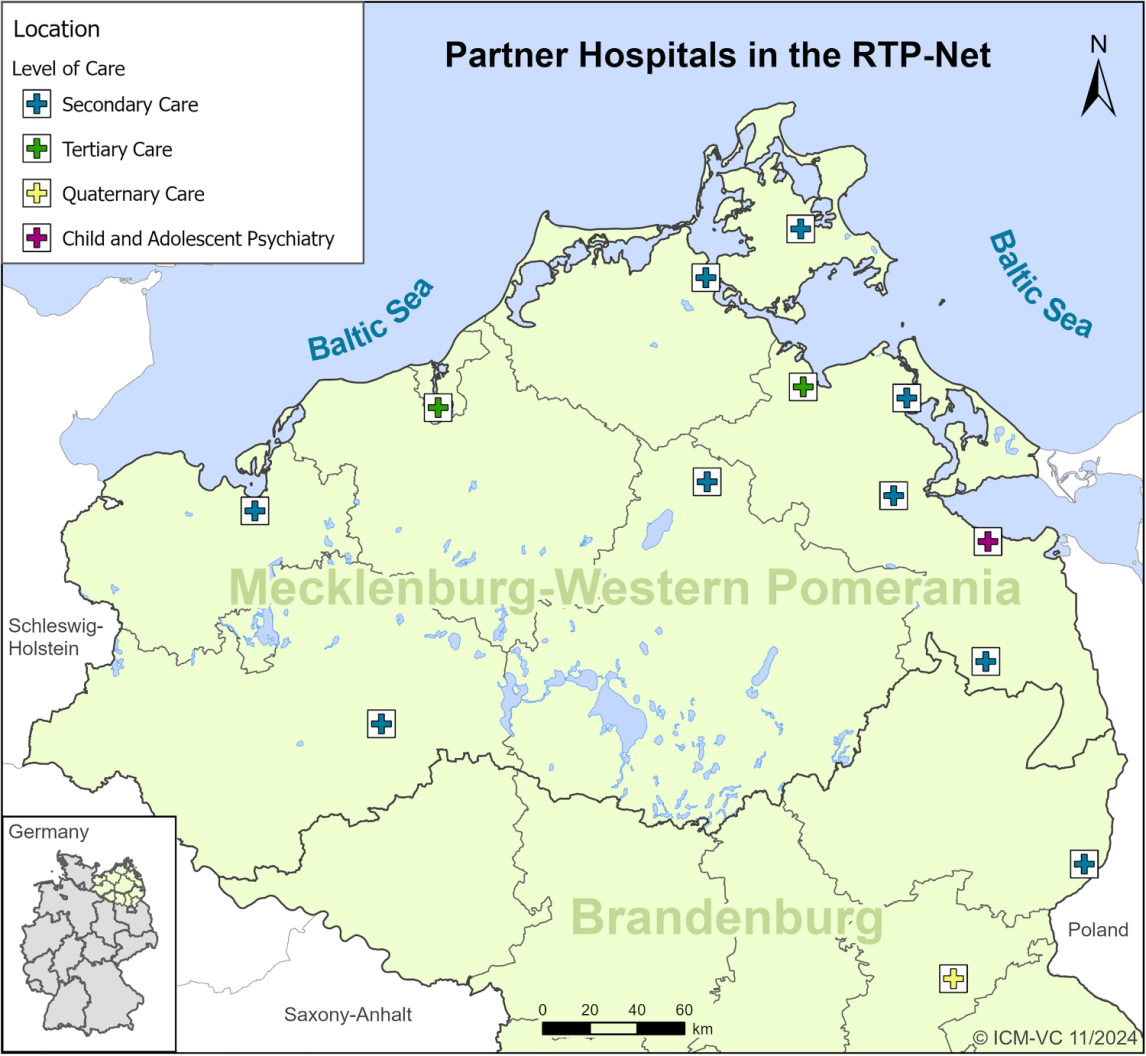
Paediatric departments from different levels of healthcare participating in the RTP-Net project.

The following study implemented a RTP-Net providing audio-visual interactive communication and digital health information exchange between paediatricians from hospitals of different sizes and specializations (see Figure 1). The study region comprises the federal states of Mecklenburg-Western Pomerania and North Brandenburg, in the Northeast of Germany.

The provided telemedical system includes tele-consultations and second opinions on medical questions in paediatric sub-specialties, like neuropaediatrics or paediatric cardiology. Moreover, paediatricians from remote provided virtual background services (rotating duty within the RTP-Net) on general paediatric or urgent questions by physicians (mainly resident physicians) in the foreground from other hospitals of the network, 24/7. The users of the RTP-Net system were free to use the telemedicine standalone or complementary to transferrals of their patients.

### Sampling

We used an open-ended snowball sampling strategy^20^ starting with the invitation of chief physicians and hospital directors from 17 hospitals with affiliated paediatric departments from Mecklenburg-Western Pomerania and Brandenburg. Then, during the first consensus conferences together with participants from 10 paediatric departments, regional patient pathways were identified that could benefit from the implementation of a RTP-Net. Afterwards, further responsible colleagues collaborating along with those pathways were identified and invited to the RTP-Net project.

The snowball sampling strategy is particularly useful for identifying key stakeholders to the implementation of a telemedical care network in a region where sub-specialized physicians are rare. In this setting, practice partners are best placed to identify eligible specialists and already existing interfacility cooperation. In addition, the participation of paediatric experts during the sampling helps to identify gaps in the regional healthcare provision that can be addressed by a telemedical networking of different hospitals. However, a snowball sampling strategy can cause a selection bias and can reduce the representativeness of the results. In order to mitigate this effect, we initially invited participants with different professional backgrounds and from different hospitals within the study region.

Criteria for the inclusion of the professionals were that they worked as a chief, specialist or resident physician in one of the participating paediatric departments that had a cooperation agreement with the project lead at the University Medicine Greifswald. Other criteria, like prior telemedicine experience or professional specializations, were not considered.

For usability testing, eligible patients were recruited for telemedicine if they were paediatric cases (age < 18 years) and were seeking care in one of the participating hospitals of the RTP-Net. The participating physicians could decide to exclude certain patients for medical reasons, like high urgency, a need for reanimation, etc. Patients were excluded from the analysis if they were older than 18 years or no tele-consultation was conducted. Patients’ prior telemedicine experiences were not considered as a criterion because telemedicine provided did not directly address patients.

Since the quantitative part of the RTP-Net study focused on descriptive analysis and was not supposed to measure an effect on a certain outcome of the telemedicine implementation, the quantitative sample was calculated based on practical considerations and not on a statistical power calculation. Practical considerations were the expected need for tele-consultations over all hospitals and the expected capacities of the paediatric departments for the recruitment of subjects. Prior to the field phase of the study, a sample size of 500 patients was calculated for the quantitative part.

### Technical infrastructure and implementation strategy

The RTP-Net system consisted of three main web-based platforms: First, an end-to-end encrypted videoconferencing web application which was used to transmit visual information on the patient health situation (e.g., remote physical examination, inspection of the setting on-site and information from the vital parameter monitor) between the telemedicine requesting hospital (TRH) where the patient was located and the telemedicine providing hospital (TPH).

Second, a web-based health information exchange system for exchanging patient health information (anamnesis, diagnosis, prior findings, etc.) was provided, the so-called eHealth-platform of the University Medicine Greifswald. The eHealth-platform consisted of a database server at the backend which is part of the secured IT infrastructure of the University Medicine Greifswald. Access to the frontend webpage of the eHealth-platform was encrypted. Only authenticated users with individual accounts and personal user certificates were able to sign in on the eHealth platform.

Third, an RTP-Net homepage was provided giving details on the RTP-Net project for internal and external stakeholders as well as linking the web applications that were relevant for the tele-consultations (arztkonsultation.de, eHealth-platform, etc.) in order to give a low threshold access to the separated telemedical applications. Moreover, a protected area of the homepage presented telemedical service descriptions and contact information about the participating paediatric departments.

For future adaptations, the RTP-Net pilot-tested also a web-based platform for tele-auscultation (emurmur.com, version 5.0.10.0, CSD Labs^®^ GmbH, Graz, Austria) and software supporting remote EEG consultations (encevis, version 2.0.4, AIT Austrian Institute of Technology GmbH, Wien, Austria). Emurmur allows sharing the signal of a digital stethoscope from the TRH with the TPH as live stream or audio recording. Encevis enables specialized neuropaediatricians to open EEG files sent by different hospitals with different file formats. This enabled the neuropediatric experts to analyse EEG data from other hospitals in a native file format and thus provide more specific recommendations.

The RTP-Net was organized in a decentralized manner, meaning that the TRH/TPH was responsible for scheduling telemedical appointments and requesting telemedical consultation. In addition, any hospital could be provider as well as customer of telemedical services since paediatricians with certain sub-specializations were not always only located at maximum care hospitals.

The telemedical intervention was designed and developed together with the clinical partners from the different participating hospitals in the RTP-Net according to the CBPR approach. The collaboration with the participants followed the CBPR-principles of building trust with community partners, iterative development of solutions and contextualizing experiences.^18,21^ The design followed an iterative, open-ended Plan-Do-Observe-Reflect cycle. The conceptional details of this approach have already been described elsewhere.^22^ The main rationale for applying a CBPR approach was that it was supposed to increase the technology acceptance of the users due to the user-centred design of the RTP-Net system. For example, some users gave us feedback that the RTP-Net system should provide an exchange of EEG data in a native file format rather than as a PDF print or via screen-sharing in order to enable neuropediatric experts to analyse the whole EEG data in-depth. However, the exchange of EEG data from various EEG devices with proprietary file formats is difficult, since the files usually cannot be re-imported by other EEG software products. That is why, we searched for a technical solution and found the encevis application (described above). After the integration of encevis in the RTP-Net system, the recruitment numbers of patients for neuropediatric tele-consultations strongly increased and the participants gave us positive feedback. Moreover, the CBPR approach allowed gaining insights into usability aspects and required adjustments of the RTP-Net system while the participants were already able to use the available telemedical services for their patients.^23^

By employing the participatory approach, the hospitals’ perspectives and ideas on the development of the RTP-Net were integrated in order to increase the acceptance of the users and to tailor the telemedicine as precisely as possible to the needs of the users and the different use cases in regional paediatric care. This participatory approach included monthly consensus meeting with the participating clinics where researchers and paediatricians were regularly discussing possible adaptations of the RTP-Net system or the dissemination of the RTP-Net system to other relevant paediatric departments in the study region. Moreover, the participants found the joint meetings useful for building trust among each other and thus became more willing to request tele-consultations from other hospitals of the RTP-Net. The participants were also involved in pre-testing the questionnaires used in this study, testing the RTP-Net system, addressing barriers to the implementation of the RTP-Net and also supported the recruitment of patients and participants for the study.

### Technology acceptance model

The analysis of the users’ technology acceptance based on the theoretical framework of the extended Technology Acceptance Model (TAM2) according to Davis and Venkatesh.^24,25^ Considering the research questions of the RTP-Net study, the main reason for choosing the TAM2 was that it is an input model that focuses on the reasons which affects the users’ intention to use a specific technology. Furthermore, TAM2 was selected since it is the most frequently used model for evaluating the technology acceptance of physicians.^26^

**Figure 2.**
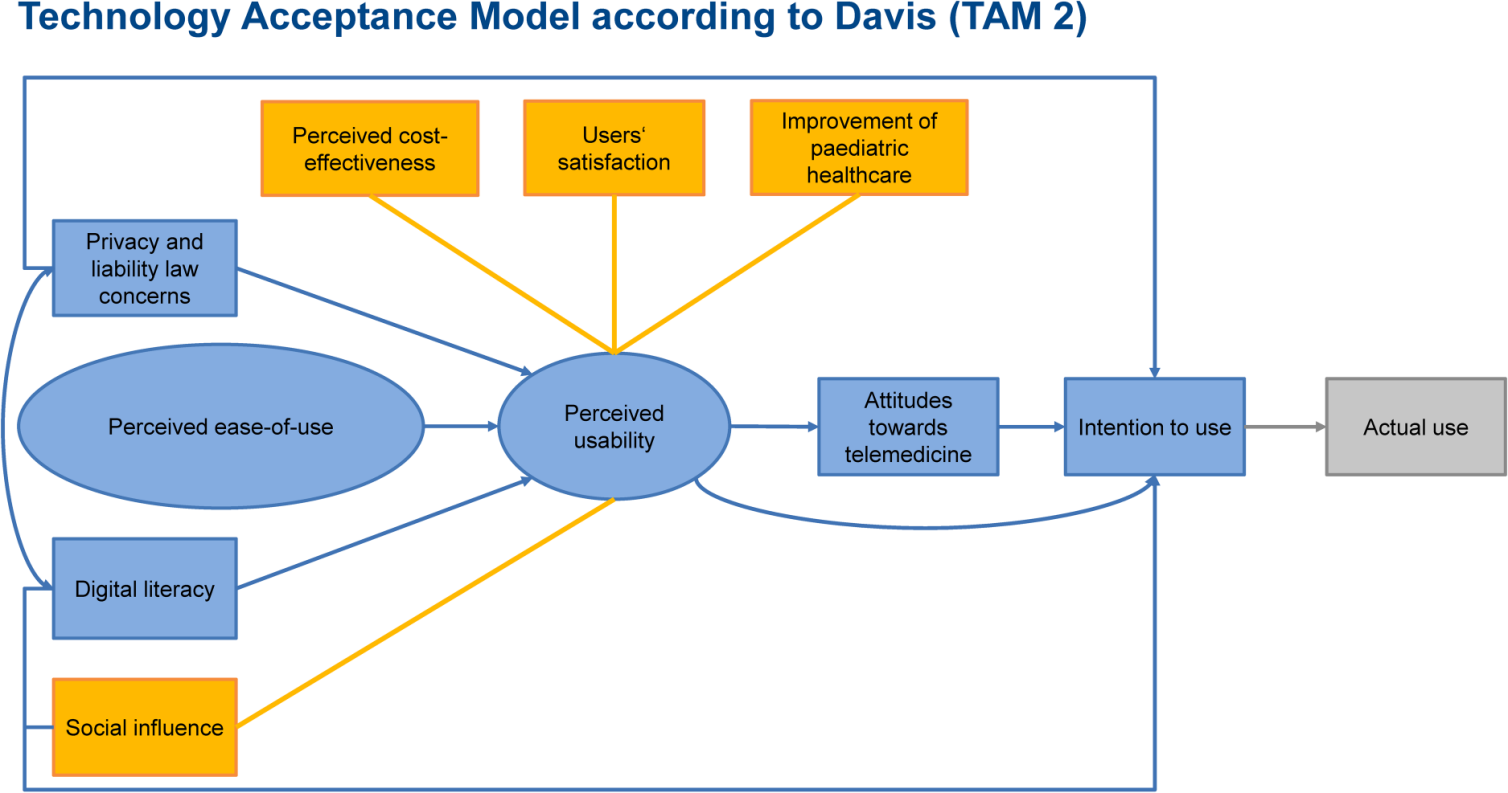
Framework of the extended technology acceptance model with adaptations to the use of information and communication technologies (ICTs) in paediatric healthcare (highlighted in yellow).

The TAM2 was adapted to the context of paediatric care and the provided telemedicine within the RTP-Net. Experiences of previous evaluations on health information exchange systems and telemedicine in healthcare settings^27^ showed that social influence, the perceived cost-effectiveness, the users’ satisfaction and expected improvements of the healthcare provision are important to the perceived usability of a technology.^22,28^ Figure 2 presents the adaptations made to the TAM2 highlighted in yellow.

Since the implemented telemedicine was used in an innovative care model at a prototypic stage, we only considered the users’ intention to use and not the actual use of the telemedicine which should be assessed after translation into usual care. An evaluation of the actual use at this prototypic stage might underestimate the actual technology acceptance of the users if the use of the telemedicine was affected by minor technical issues that typically occurred in the prototypic stage of a technology. The intention to use was analysed quantitatively with a questionnaire (see the section on quantitative data collection and analysis below).

All other components of TAM2 guided the qualitative analysis, respectively, the deductive content analysis of the project diary (see the next section on the qualitative part of the data collection and analysis).

### Quantitative part

#### Data collection and analysis

After each telemedical consultation, the involved physicians of the RTP-Net were asked to fill out an electronic questionnaire to assess the users’ satisfaction with the RTP-Net system (5-point Likert scale), their intention to use the system in the clinical practice (dichotomous question) and technical issues that occurred during the tele-consultations. In case the respondents indicated that they do not perceive the RTP-Net system adequate for supporting clinical practice, a free-text question asked for the reasons. The questionnaire was pretested by three paediatricians and one anaesthesiologist from different hospitals participating in the RTP-Net project before the field phase started. The questionnaire used in this study is attached as a supplementary file.

The data was recorded with electronic case report forms in the eHealth platform, between January 2021 and March 2024. We used descriptive statistics for analysing the data. The analyses were performed in SAS^©^ Enterprise Guide, ver. 8.3 (SAS Institute, Cary, USA).

### Qualitative part

#### Data collection and analysis

An open-ended participant observation approach was applied to observe aspects of the RTP-Net system that were relevant to the users’ technology acceptance as well as barriers and facilitators to the implementation.^29^ After each encounter or contact between the research group and the representatives of the participating hospitals, for example, during workshops, visits in the hospitals and individual contacts via email or phone, the researchers recorded the content of the contact as well as the time, place, type of contact and the involved representatives of hospitals in a project diary. The records were conducted in a semi-structured way due to the explorative design of the RTP-Net study. The recorded project dairy entries were eligible for the analysis if the content referred to implementation facilitators or barriers, or to technology acceptance aspects of the RTP-Net system mentioned by the participants.

**Figure 3.**
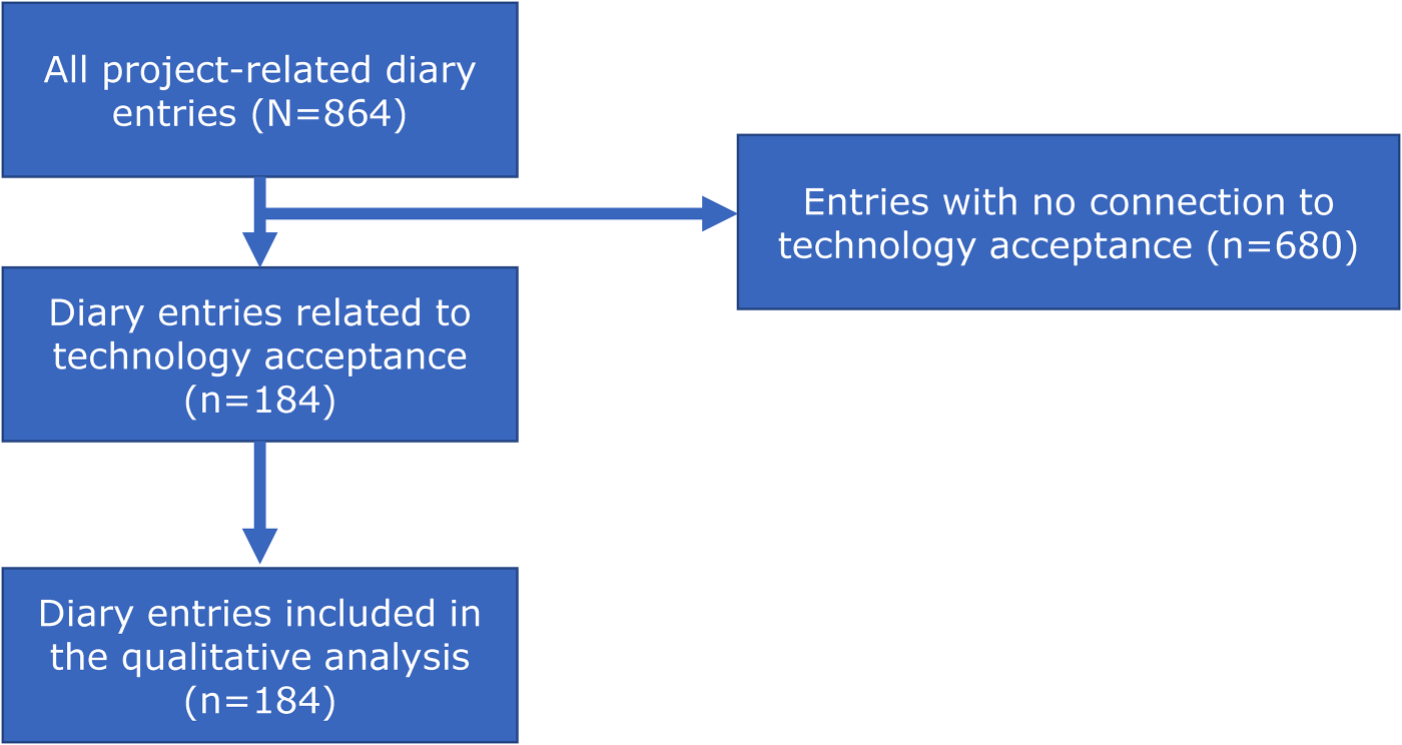
Flowchart of the eligible diary entries related to technology acceptance aspects.

The joint consensus conferences with the participating hospitals were conducted monthly. The frequency of other contact types varied and based on the need for discussing or clarifying issues of the implementation. As shown in the flowchart in Figure 3, a total of 864 contacts/interactions were documented, whereas 184 contacts considered aspects of the users’ technology acceptance. Qualitative data was collected between April 2020 and March 2024.

Finally, at least one other researcher checked the project diary entries for inconsistencies before starting with the qualitative analysis.

For the qualitative analysis, we used a deductive-inductive content analysis which was conducted independently by two researchers. Themes were initially developed deductively based on the components of the adapted TAM2 model (see Figure 2) and were expanded inductively during the analysis of the project diary entries if new aspects of the themes arose. Furthermore, descriptive statistics were used in order to analyse the frequencies of codings in the corresponding code category or in relation to specific document variables. The qualitative data was analysed with MAXQDA^©^ 2022 (VERBI Software – Consult -Sozialforschung GmbH, Berlin, Germany). MAXQDA supports validating and organizing the qualitative data by enabling different persons to work together on the categorization, coding and analysis. It also supports mixed-methods analysis of qualitative date.

## Results

### Sample

Table 1 shows the characteristics of the paediatric departments participated in the RTP-Net and the telemedical services they provided or utilized.

**Table 1.** Characteristics of the participating hospitals and their roles in the RTP-Net.

Hospital location	Healthcare level	Provided telemedical services	Utilized telemedical services	Participating physicians (N, %)
Anklam	SC	Virtual background services	Virtual background services Specialized tele-consultations	3 (2.13%)
Bergen	SC	Specialized tele-consultations in allergology	Specialized tele-consultations	14 (9.93%)
Demmin	SC	Virtual background services	Virtual background services Specialized tele-consultations	13 (9.22%)
Eberswalde	QC	Specialized tele-consultations in diagnostics and treatment of vascular malformations Neonatology	Virtual background services	3 (2.13%)
Greifswald	TC	Virtual background services Specialized tele-consultations in: Neuropaediatrics Neonatology Paediatric intensive care and emergency care Oncology and haematology Rheumatology Nephrology Cardiology Endocrinology	Virtual background services	40 (28.37%)
Pasewalk	SC		Virtual background services Specialized tele-consultations	2 (1.42%)
Parchim	SC	Virtual background services Specialized tele-consultations in neonatology	Virtual background services Specialized tele-consultations	5 (3.55%)
Rostock	TC	Virtual background services Specialized tele-consultations in: Paediatric gastroenterology Pneumology Oncology Paediatric palliative care Radiology	Virtual background services	37 (26.24%)
Schwedt	SC	Virtual background services	Virtual background services Specialized tele-consultations	4 (2.84%)
Stralsund	SC	Virtual background services Specialized tele-consultations in cardiology	Virtual background services Specialized tele-consultations	8 (5.67%)
Ueckermünde	SC	Specialized tele-consultations in child and adolescent psychiatry		1 (0.71%)
Wismar	SC	Virtual background services	Virtual background services	8 (5.67%)
Wolgast	SC	Virtual background services Specialized tele-consultations in paediatric oncology	Virtual background services	3 (2.13%)

SC: secondary care level; TC: tertiary care level; QC: quaternary care level.

As can be seen from Table 1, secondary care level hospitals not only requested telemedical support, but also provided virtual background services or even specialized tele-consultation in certain paediatric sub-specializations.

#### Survey participants

From the above-mentioned hospitals (see Table 1), 26 TPH physicians and 30 TRH physicians conducted tele-consultations and participated in the survey. In total, the users from both sides (telemedical clinic and clinic on-site) filled out 907 questionnaires. The TPH physicians filled out 483 (response rate: 483/507 = 95.27%) and TRH physicians 413 (response rate: 413/507 = 79.49%) questionnaires. 11 times the information about who filled out the questionnaire was missing. Table 2 shows how often physicians with different position participated in the tele-consultation from either the TPH or TRH.

**Table 2.** Positions of the physicians from the telemedicine requesting or providing hospital participating in the tele-consultations (*N* = 907 with 16 missing data).

Position	Telemedicine providing hospital		Telemedicine requesting hospital		In total	
	*N*	%	*N*	%	*N*	%
Chief physicians	331	69.1	148	35.9	479	53.8
Senior physicians	103	21.5	7	1.7	110	12.3
Assistant physicians	45	9.4	257	62.4	302	33.9
Total	479	100.0	412	100.0	891	100.0

Between January 2021 and March 2024, the participating physicians recruited 431 patients. *N* = 4 patients were excluded since they were older than 18 years and *n* = 24 patients were excluded since no tele-consultation was provided. It is assumed that the exclusion of patients without tele-consultation from the analysis should not have had an impact on the analysis, since in all cases the hospitals reported that the omission of tele-consultation after the admission of the patient to the study was due to medical reasons and not due to the RTP-Net system. The included *n* = 403 patients received 507 tele-consultations via the RTP-Net. In 45 of these cases, no satisfaction questionnaire was filled out. Table 3 shows the patients’ characteristics.

**Table 3.** Characteristics of the included patient (*N* = 403).

	Female (*n* = 198; 49.1%)	Male (*n* = 205; 50.9%)	In total (*n* = 403; 100.0%)
Age (in years)			
Mean	7.01	6.64	6.82
SD	5.56	5.78	5.67
Most important Diagnosis Groups (ICD 10)			
G – diseases of the nervous system			
N	41	37	78
%	24.0	20.3	44.3
R – Symptoms and signs of diseases			
N	35	44	79
%	20.5	24.2	44.7
J – Respiratory disorders			
N	20	27	47
%	11.7	14.8	26.5

#### Participants in the qualitative analysis

Table 4 provides an overview about the physicians who participated in the qualitative part of the RTP-Net study, sorted by their gender and positions.

**Table 4.** Number of physicians (*N* = 141) who participated in the qualitative part of the RTP-Net study regarding their gender and position in the respective hospital.

	Chief physicians, *N* (%)	Specialist physicians, *N* (%)	Resident physicians, *N* (%)	In total, *N* (%)
Male, *N* (%)	10 (52.63%)	10 (38.46%)	26 (27.01%)	46 (32.62%)
Female, *N* (%)	9 (47.37%)	16 (61.53%)	70 (72.99%)	95 (67.38%)
In total, *N* (%)	19 (13.55%)	26 (18.44%)	96 (68.01%)	141 (100.00%)

The sample of the qualitative part was representative with respect to thematic saturation. Achieving thematic saturation was defined by: a) Including the perspectives of at least one chief physician, and one specialist physician or resident physician from each of the participating hospitals, and b) thematic saturation is achieved when repetitive codes or themes are identified across the participating hospitals.

### Quantitative results: Users’ satisfaction and intention to use

#### Satisfaction of the users

**Figure 4.**
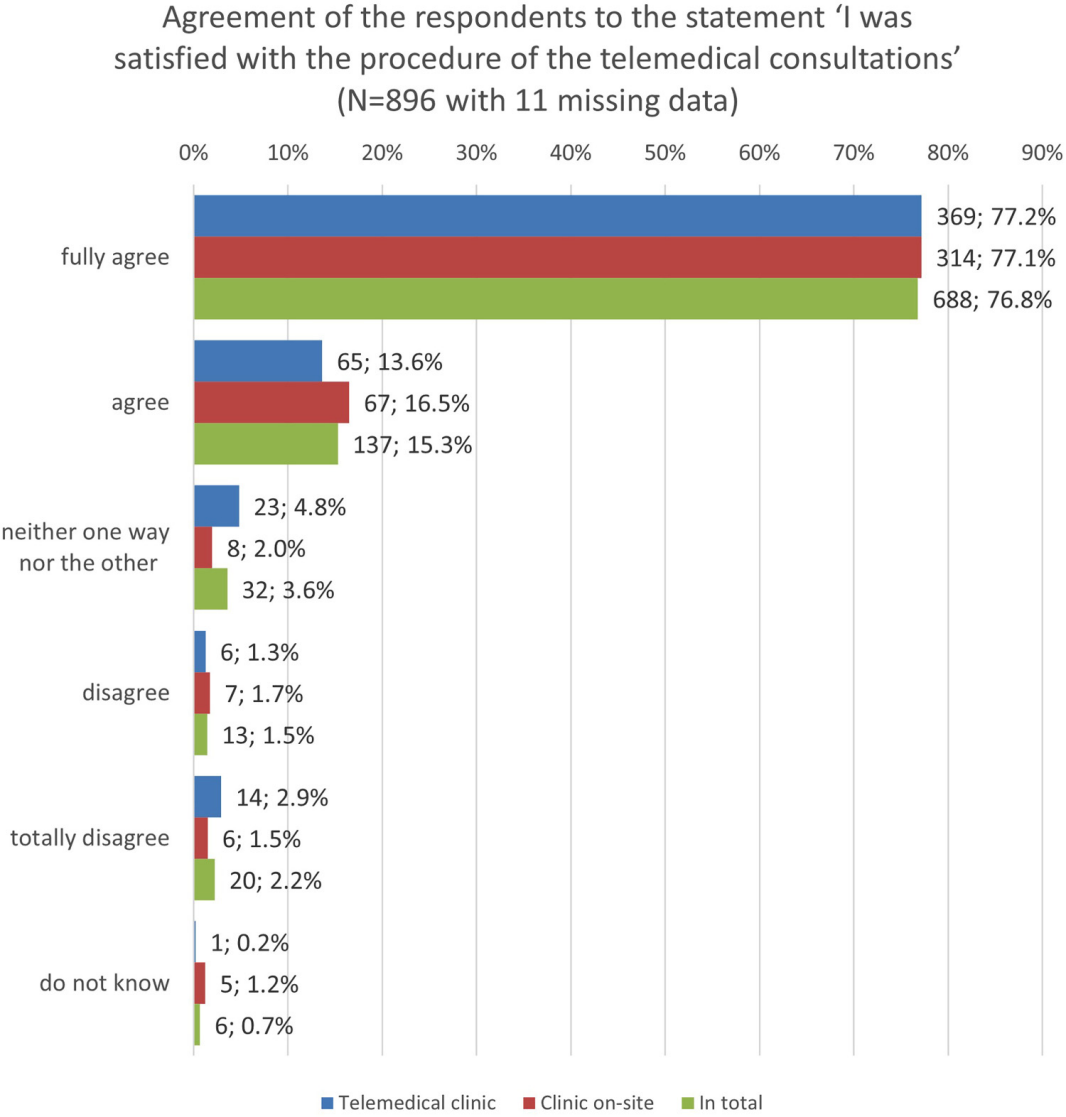
Satisfaction of the users with the RTP-Net system (*N* = 896 with 11 missing data).

The quantitative results show high satisfaction scores of the users with the conducted tele-consultation (Figure 4).

The overall agreement and, especially, the agreement between the TPH and the TRH is interesting since it shows that the RTP-Net system supports sufficiently the clinical decision-making in the TRH as well as the ability of the TPH physician to assess the health situation of a patient from remote.

#### Intention to use

The participating physicians found the tele-consultation adequate to discuss the patients’ health situation in 95.83% (*n* = 850/887; missing data = 20) of the conducted tele-consultations. This shows that the users’ intention to use the RTP-Net system is high.

**Figure 5.**
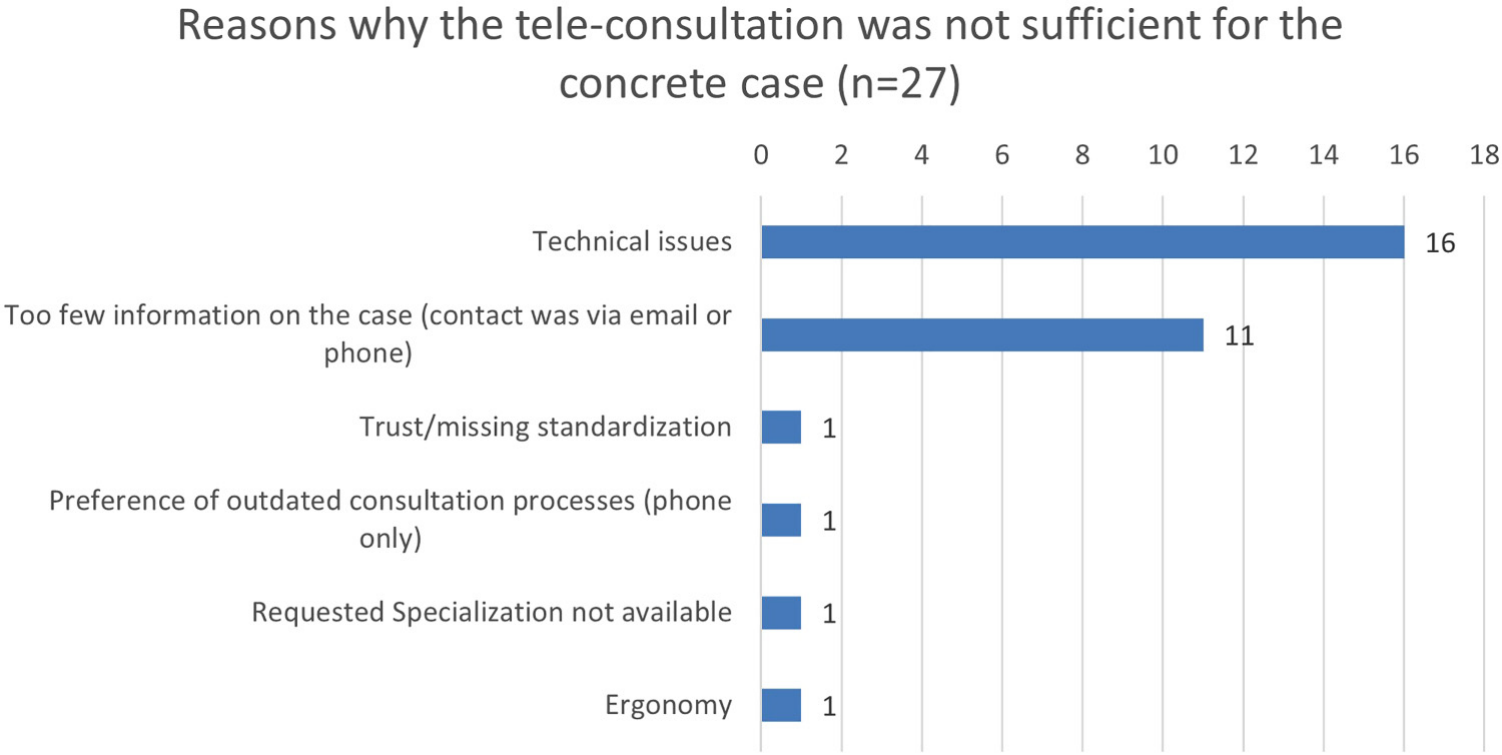
Reasons why the paediatricians did not think that the tele-consultation was suitable for supporting usual paediatric care (in numbers).

The physicians could document reasons why they did not consider the RTP-Net system to be sufficient for supporting usual paediatric care (see Figure 5).

It can be seen from the data in Figure 5 that technical issues were the most frequent reason for users to reject the use of the RTP-Net system.

#### Technical issues

**Figure 6.**
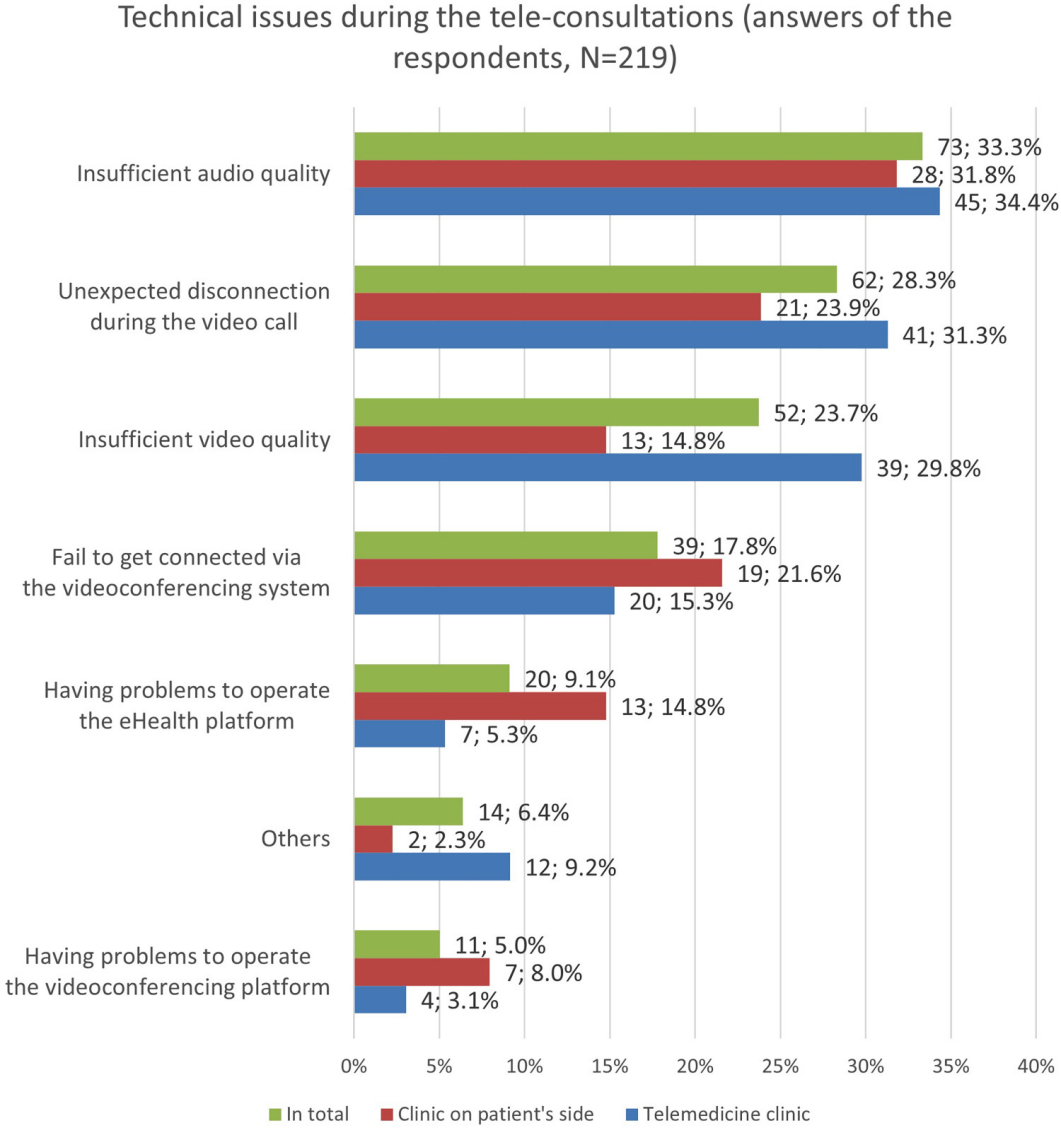
Technical issues occurred during the tele-consultations as reported by the telemedicine physicians or/and the physicians on the patient's end. Multiple choices were possible.

The physicians on the telemedicine side experienced technical issues during 29.4% of the consultations (*n* = 141/480; missing data: 3). The physicians at the patient's side noticed technical problems during 22.6% (*n* = 96/412; missing data: 1) of the tele-consultations. Further 11 answers to the question if there were occurring technical issues during the use of the telemedicine were excluded from this analysis, since it was not clear which side had filled out the questionnaire. Figure 6 shows in detail the technical issues which occurred during the tele-consultations.

The most often reported issues, bad audio and video quality as well as disconnection during the videoconference, were often related to varying internet connectivity or interferences between the provided web portal and local firewall set-ups of individual hospitals. It is noticeable that the TPH physicians reported insufficient audio and video quality more often than the TRH physicians. Interestingly, the overall frequency of technical issues during the tele-consultation (almost one-third) contrasts the high satisfaction and intention-to-use rates of the users. Surprisingly, the technical issues seem barely affecting the users’ technology acceptance of the RTP-Net system.

The participants reported only very few non-technical issues. In total, during 1.60% (*N* = 14/877, missing data = 30) of the tele-consultations non-technical issues occurred. The participants mentioned time-restraints and negative effects on the patient–doctor relationship as examples for non-technical issues that accompany the use of telemedicine.

### Qualitative results: Technology acceptance regarding the RTP-Net system perceived usability of the RTP-Net system

#### Improvement of paediatric healthcare

Table 5 shows the users’ perceptions of the usability of the RTP-Net in terms of effects on the healthcare provision.

**Table 5.** The users’ perceptions of the usability of RTP-Net system (*N* = 69).

Positive perceptions of the usability (60.8%, *n* = 42)	Negative perceptions of the usability (39.2%, *n* = 27)
Improved availability of patient health information (26.2%; *n* = 11) Improved access of patients to specialized healthcare providers, especially of patients living in rural areas (23.8%; *n* = 10) Quickly available second opinion (21.4%; *n* = 9) Remote support and training of resident physicians by experienced colleagues (21.4%; *n* = 9) Others (7.1%; *n* = 3): improved patient adherence (*n* = 1), possibility to follow-up the patient's further treatment after transferral (*n* = 2)	Interoperability issues and lacking digital infrastructure hinders the exchange of patient findings in all different kinds of file formats (44.4%; *n* = 12) The system lacks features in order to support acute emergency care properly (33.3%; *n* = 9) The decentralized organization and coordination of the telemedicine within the RTP-Net by the providers of the telemedical services hinders an effective telemedical care (7.4%; *n* = 2) Examinations that need to be performed in person (7.4%; *n* = 2) Others (7.4%; *n* = 2): concerns about the patient safety (*n* = 1), examinations that need to be performed in person (*n* = 2), lack of resources (*n* = 1)

The majority of participants highlighted positive usability aspects of the RTP-Net, like an improved availability of treatment information and easier access to second opinions of colleagues as well as an improved patients’ access to paediatric specialties which aligns with high the user satisfaction scores found in the quantitative results.

An example of this was the presentation and discussion of cases with unclear seizures from smaller hospitals with a neuropediatric expert via the RTP-Net. The TRH physicians highlighted that, due to the RTP-Net system, further diagnostics and treatments recommended by the neuropediatric expert could be conducted on-site without the need to refer the patients and their parents to the next specialized hospital which usually takes month to receive an appointment and also would have meant longer travel times to and waiting times at the specialized hospital.

However, the relatively high number of negative perceptions mentioned contrasts the high user satisfaction scores found in the quantitative data. An explanation for this contradiction could be that, during the qualitative consensus conferences, the participants were rather focused on the necessary adaptations of the RTP-Net system and, during the quantitative prototype testing, they experienced the useful aspects of the system for overcoming barriers in regional paediatric care.

#### Perceived cost-effectiveness

The users perceived the effort and resources needed to use the RTP-Net system differentially. Table 6 presents the different positive and negative arguments as well as the corresponding numbers of coding in the project diary.

**Table 6.** Estimated effort and resource requirements of the RTP-Net in the perceptions of the users (*N* = 31).

Positive arguments (48.4%; *n* = 15)	Negative arguments (51.6%; *n* = 16)
More efficient use of healthcare resources (33.3%; *n* = 5) No reimbursement of telemedicine in the status quo, but in the RTP-Net project (26.7%; *n* = 4) Less documentation effort (20.0%; *n* = 3) Reduction of time and transportation costs (20.0%; *n* = 3)	Lack of resources for the implementation and use of the RTP-Net system (75.0%; *n* = 12) High documentation effort (18.7%; *n* = 3) High effort for obtaining a patient's informed consent (6.3%; *n* = 1)

A likely explanation for the users’ mixed assessments regarding the potential of the RTP-Net to reduce the workload of the paediatric departments may be that the implementation of the RTP-Net system was initially more complex in some hospitals than in others due to local administrative barriers or disruptive IT settings. In addition, the use of the RTP-Net system involved new documentation tasks, which were useful but also required additional work for the participants compared to the status quo ante. Therefore, maybe some participants reported a higher documentation effort due to the use of the RTP-Net system. However, other participants reported a relief of their departments since some unnecessary patient referrals could be prevented due to the use of the RTP-Net system.

#### Users’ satisfaction

The users’ overall satisfaction with the RTP-Net was high. High satisfaction with the RTP-Net was expressed 19 times (83%, *n* = 19/23), low satisfaction 4 times (17%, *n* = 4/23) and confirmed the high satisfaction scores found in the quantitative findings.

#### Perceived ease-of-use

**Figure 7.**
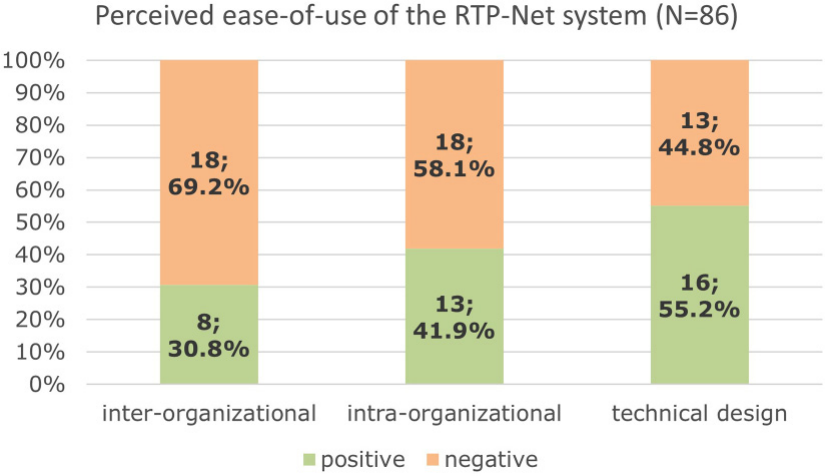
Perceived ease-of-use of the RTP-Net system (*N* = 86), subcategorized and sorted by positive and negative ease-of-use aspects. Presented is the proportion of positive and negative coding in each subcategory.

The perceived ease-of-use codings was assigned to three subcategories (see Figure 7):

1. Inter-organizational ease-of-use refers to the overall organization of the RTP-Net system, like the ease of providing and utilizing telemedical services via the RTP-Net system.
2. Intra-organizational aspects refer to parameters that affect the ease-of-use of the RTP-Net within a certain clinic, such as the internet connectivity, local firewalls and other local aspects that affected the performance or integration of the RTP-Net system.
3. The subcategory ‘Technical Design’ refers to ease-of-use aspects related to the provided devices and software of the RTP-Net.

Overall, the ease-of-use of the RTP-Net system yielded mixed results. The technical design seems to be less critical to the ease-of-use than the intra- and inter-organizational issues. Inter-organizational issues seem to be the most challenging to the ease-of-use of the RTP-Net in the perspective of the users. An instance for this is that some participants found it too complex to schedule appointments for tele-consultations via the RTP-Net or to timely find the right specialist physician for a specific medical question at the TPH of the RTP-Net.

#### Digital literacy of the users

**Figure 8.**
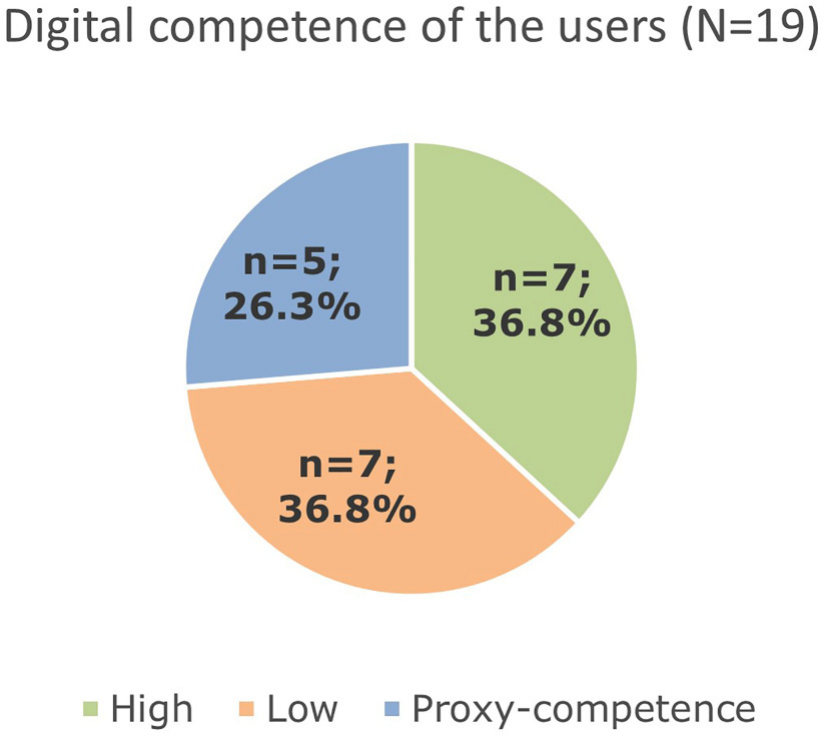
Frequency of coding statements categorized to high, low user satisfaction or proxy-competence (*N* = 19).

Figure 8 shows the digital literacy of the users.

The majority of the participants in the RTP-Net had a low digital literacy or had to involve other colleagues helping them to use the RTP-Net system (proxy-competence).

#### Privacy and liability law concerns

The participants mentioned legal concerns with respect to telemedicine 16 times in total (100.0%; *N* = 16), thereof privacy concerns 10 times (62.5%) and liability concerns 6 times (37.5%). However, the RTP-Net was often (62.5%; *n* = 10/16) seen as an improvement in terms of liability and privacy protection compared to the status quo ante, since most of the participants had cooperated informally with each other until the implementation of the RTP-Net system.

The participants also saw concrete benefits in the use of the RTP-Net, like the mandatory documentation of the tele-consultations within the RTP-Net system after each consultation in a short report. This made the TPH physicians feel more confident in making recommendations to other colleagues, as the decision-making process became transparent and traceable.

Three times (18.8%), participants raised liability and privacy concerns directly with respect to the RTP-Net. The reason was in all cases that the telemedicine doctors were concerned about giving telemedical consultations to physicians from other clinics with unknown qualifications and experiences in paediatrics. This is why the TPH physicians wished for inter-organizational guidelines and standards in the telemedical treatment and diagnostics of children, since this would ensure a standardized and uniform transmission of medical parameters to the TPH as well as a professional implementation of treatment recommendations by the TRH physicians. As a consequence, the participants standardized the anamnesis for the virtual background service. A corresponding anamnesis form was integrated into the eHealth platform that should guide resident physicians in the foreground to collect all relevant anamnestic information in a standardized format (e.g., which metrics should be used to report blood pressure measurements and so on).

#### Social influence

Further analysis showed that 28 times (100.0%; *N* = 28) the users mentioned that social influences affected their technology acceptance. We categorized the social influence into the following sub-categories:

1. Colleagues from other organizations than the users’ affiliated organization recommended the use of RTP-Net (32.2%; *n* = 9).
2. Advice to use the RTP-Net by supervising physicians of the same hospital (32.2%; *n* = 9).
3. Trust within the RTP-Net (21.4%; *n* = 6).
4. Colleagues of the same hospital recommended the use of RTP-Net (14.2%; *n* = 4).

The influence of colleagues from other organizations (*n* = 9) affected the users technology acceptance of the RTP-Net mostly positive (89.0%; *n* = 8/9), for example, TPH physicians suggested TRH physicians to use the RTP-Net in order to provide more complete patient data and in turn receive more precise medical recommendations for their patients. The influence of supervising physicians and other colleagues affected the technology acceptance of the users less often positively (supervisors’ positive effect: *n* = 4/9; 44.4%; effect of direct colleagues: *n* = 2/4; 50.0%). The participants considered trust among the RTP-Net users to be important for the use of the RTP-Net system.

#### Attitudes towards RTP-Net

Different user attitudes towards the RTP-Net system (*N* = 48) were identified, which had a positive or negative impact on the users’ technology acceptance (see Table 7).

**Table 7.** User attitudes affecting the users’ technology acceptance of the RTP-Net system.

Positive attitudes towards the RTP-Net (62.5%; *n* = 30)	Negative attitudes towards the RTP-Net (37.5%; *n* = 18)
Motivation to bring in own resources or time (50.0%; *n*= 15) Addressing technical or organizational barriers within the same organization or hospital (36.7%; *n* = 11) Others (13.3%; *n* = 4)	Too positive expectations regarding the provided technologies (55.6%; *n* = 10) Lack of engagement (22.2%; *n* = 4) Scepticism towards telemedicine in general (16.7%; *n* = 3) No perceived progress of the project (5.5%; *n* = 1)

We observed that some participants were more motivated than others to spend time and resources for the project and that this seemed to affect the acceptance of the RTP-Net. For example, some departments assigned one or more physicians to accompany the implementation process of the RTP-Net system on-site. The responsible physicians were, for example, available for questions from their colleagues, reported technical issues to the project team or coordinated the tele-consultations with other hospitals.

However, excessively high user expectations, which the RTP-Net system could not meet, led to frustration and lower acceptance. For example, one TPH physician would have preferred the integration of augmented reality glasses, which was not yet possible at the time of the technological development.

#### Identified useful adaptations and extensions of RTP-Net

Throughout the course of implementing the RTP-Net, we identified together with the participating physicians additional use cases and potentials for further development. Tele-auscultation and Tele-EEG were identified as potentially useful adaptations very early in the project. So, we already implemented functionalities for Tele-EEG and Tele-auscultation in 2023 to test their feasibility.

Further potentially useful adaptations from the perspective of the users included: remote palliative care of paediatric patients, telemedical support for forensic medicine in paediatrics, extension of the RTP-Net system by adding smart devices such as smart glasses supporting augmented reality, inter-disciplinary tele-consultations with other medical specialties, for example, between paediatrics and dermatology, telemedical assisted transportation of critically ill children to the next specialized hospital or paediatric ICU, remote consultation with the patients and parents before admission or after discharge.

### Discussion

#### Summary of the principal results

In summary, the results show that the implementation of a RTP-Net faces some barriers, like interoperability issues between the different participating clinics and their local IT systems, a need for technical adaptation to certain use cases (e.g., emergency care or remote advanced telemedical diagnostics) and a more efficient organization of telemedical requests and services. Moreover, there are some individual characteristics of the participants that hinder an effective implementation, such as scepticism towards telemedicine, a favouritism of traditional communication means and processes to which the users have used to, and varying digital competences among the users.

The study observed that a tele-paediatric network is widely accepted by hospitals and physicians and it is perceived as useful in order to support the paediatric care in rural regions. The users’ satisfaction and intention to use scores were high, but also contrasted by a high frequency of technical issues reported and users’ scepticism towards the implementation of the RTP-Net in a usual care setting.

#### Research question 1: Effectiveness of the implementation of the RTP-Net

An initial objective of the study was to evaluate how effective the implementation of the RTP-Net was in terms of the users’ technology acceptance and satisfaction. The quantitative results confirmed the effectiveness of the implementation in some of the explored use cases and for certain reasons. The qualitative content analysis based on the TAM2 components revealed the reasons why the RTP-Net was in some cases more effectively adopted than in others.

#### Research question 2: Facilitators and barriers to the implementation of the RTP-Net system?

The second question in this research was what aspects of the RTP-Net system act as facilitators or barriers to its implementation and use. What follows is a discussion of implementation facilitators identified:

One interesting finding was that the children's access to specialized services can be improved by the use of RTP-Net in rural regions where the paediatric departments had to reduce their healthcare services over the recent years. This finding is consistent with that of a systematic review on telemedicine in paediatric emergency care^30^ also concluded that telemedicine improves the access to subspeciality expertise for children living in locations without in-person subspeciality access and that telemedicine reduces the need to transfer of critically ill children. Especially for neuropediatric care, the qualitative data showed that the patients’ access was improved by tele-consultations via the RTP-Net system which is in line with retrospective cohort study that showed that less transferrals of children were necessary due to the use of tele-consultation between primary care physicians and the tertiary care hospital with a neuropediatric expert.^31^ Another study could also show that a telemedical neuropediatric network could increase and sustain the patients’ satisfaction in children living with epilepsy in remote areas of Brasil.^32^

Another important finding is that physicians from the participating hospitals in rural regions highlighted the timely availability of a specialist's (second) opinion and his or her support in the diagnosis or treatment of children on-site. This is important since it indicates that the use of RTP-Net improves the timeliness of care without discontinuances. Continuity of care is recognized as a cornerstone for the timely identification of chronic conditions with the risk for childhood onset as well as the personalized care of these conditions.^33^

Another finding that stands out from the results reported earlier is that the tele-paediatric network allows a knowledge transfer between specialized centres and smaller hospitals. This makes it for resident physicians in training more attractive to work in smaller hospitals as they have the opportunity to discuss complex cases with a specialist or get the chance to follow-up cases that they had to transfer to a specialized hospital. Against the background of labour shortages in paediatrics, this aspect of RTP-Net could contribute to the promotion and qualification of junior paediatricians in rural regions.

Perhaps the most compelling finding is that the tele-consultations via RTP-Net support clinical decision-making by providing more detailed medical information than is the case in the status quo when physicians consult specialist physicians solely by phone. However, extending tele-consultations to include telemedical diagnostics (e.g., tele-auscultation, tele-EEG, tele-sonography) could broaden the scope of application of the RTP-Net.

Controversial facilitators identified were the users’ attitudes to telemedicine in general, the perceived cost-effectiveness and the social influence by other physicians. In contrast to Shi et al.,^34^ our study found not that a ‘hedonic motivation’ of the users leads to an underestimation of medical safety aspects. On the contrary, early adopters in the RTP-Net found that the use of the telemedical system improved the medical safety in comparison to the status quo ante, which often means unscheduled phone calls with less clinical and unstructured clinical information and a lack of documentation regarding the conducted phone consultations. So, the motivation to go first with the use of the telemedical system was a result of the urge to improve the medical safety and to improve the healthcare of children especially in rural regions where children sometimes are not seen by a paediatrician at all.

The cost effectiveness of the tele-consultations was seen positive within the project, apparently due to the expense allowance paid by the RTP-Net project for each case, and promoted the implementation of the RTP-Net. However, the users expected the cost effectiveness of the RTP-Net could be insufficient under the conditions of the reimbursement schedule in the real healthcare system that considers telemedical services not enough. A cross-sectional study on telemedicine acceptance in Chinese paediatric clinics also came to the conclusion that hospitals and their paediatricians are less willing to adopt telemedical services if they perceive the reimbursement of services as insufficient.^34^

Moving on now to consider the implementation barriers identified:

Interestingly, participants that were more sceptical about the use of telemedicine in general also preferred outdated or less secure communication means like phone calls or emails. This finding suggests that some physicians rather prefer communication means which they used to than digital means which require time and practice to implement. It could be argued that due to labour shortages and heavy clinical workloads, paediatricians are often unable to devote the time required to implement telemedicine.^13^ One solution for this barrier might be the implementation of national/federal authorities with their own competences in regulating telemedicine and incentivizing the implementation and use of telemedicine in healthcare facilities.

Surprisingly, some participants had legal concerns about the use of telemedicine, which sometimes hindered an effective implementation of the RTP-Net system. However, before the implementation of the RTP-Net, a medical lawyer provided the participants with a report on liability and privacy aspects of conducting tele-consultations in Germany. The medical lawyer also assisted the participants with legal consultations throughout the project. Moreover, the provided platforms included the necessary data protection and privacy safeguards. A possible explanation for the remaining legal concerns reported by some participants could be the missing trust of the users in the safety of the provided system and in the physicians participated from external departments. The availability of a nationwide certified and released technical infrastructure as a basis for telemedical applications and federal telemedicine centres with specialist physicians designated to specific telemedical requests could create the trust needed.

In contrast to the high users’ intention to use and satisfaction scores, during about one-third of the conducted tele-consultations, technical issues occurred and were perceived as most important barrier to the use of the RTP-Net system. One possible explanation for this contradiction might be that paediatricians from rural healthcare settings already faces immense challenges in the treatment of children with a need for specialized care and, therefore, urgently rely on interfacility cooperation and health information technologies supporting such collaborations regardless of minor technical issues they might have. This explanation is supported by the observation made in the RTP-Net project that most of the participants were less interested in testing the RTP-Net system with test subjects than with real patients who actually needed a specialist physicians’ opinion.

Furthermore, technical issues, such as bad audio or video quality, seem to affect telemedicine providing paediatricians more than the paediatricians at the patient's side. The reason for that might be that the telemedicine physicians depend more than the requesting site on good audio and video quality in order to support adequately clinical decision-making. This is consistent with a previous studies that have noted the importance to address technical issues of telemedicine, for example, by conducting frequent user training, providing a suitable infrastructure, integrative system interfaces, sufficient video and audio quality and a good network signal and transmission speed.^34^ In addition, the RTP-Net study found that technical assistants or individuals with a high digital literacy supporting their colleagues on-site fostered the adoption of the RTP-Net system. The early identification and involvement of these promoting persons in implementation processes might help to implement more efficiently telemedicine in understaffed healthcare facilities.

Besides technical issues, in some cases, the provision of incomplete patient health information to the telemedicine physician was one of the most often documented barriers to the use of the RTP-Net system. The findings reported here suggest the need for a comprehensive and interoperable digital infrastructure as basis for telemedical applications in Germany.^35^ Even though the eHealth act (in German E-Health-Gesetz) in 2015,^36^ regulated the development of a safe and interoperable nationwide digital infrastructure for the German healthcare system (in German Telematikinfrastruktur) the digitalization of the German healthcare system still lags behind other European and international countries. In 2020, the German Government passed the so-called Hospital Future Act (abbreviated in German as KHZG)^37^ which finances digitalization projects and obliges hospitals to digitalize their workflows and processes, such as implementing digital patient portals, digital medication management systems and digital documentation systems for medical and nursing services. However, the process of the digitalization is still ongoing and the due date for the hospitals to commission IT providers of health information technologies has been recently extended to the end of 2025 because of the nationwide delay in the implementation progress.

In 2024, the new German Digital Act (in German Digital-Gesetz)^38^ came into force and set up a new authority (Competence Centre for Interoperability in the Healthcare System, in German Kompetenzzentrum für Interoperabilität im Gesundheitswesen). This Competence Centre has extensive powers and involves various representatives of the healthcare system in order to identify and prioritize practical needs which might accelerate the implementation of digital health information exchange and telemedicine in the German healthcare system. The task of the Competence Centre is also to regulate binding interoperability standards as well as data protection and privacy safeguards, and to certify primary systems that fulfil the requirements. In addition, the legal responsibility for the safety of the provided health information technologies is delegated to the Competence Centre which could increase the trust of the healthcare providers in the use of telemedicine, as discussed above.

However, it is not clear yet if and how the recent regulations will consider the specific challenges and needs of paediatric healthcare for rural-dwelling children and adolescents. The recently founded German Centre for Children and Adolescent Health as well as initiatives of the Federal State of Mecklenburg-Western Pomerania point in the right direction. In 2024, the Federal State of Mecklenburg-Western Pomerania published a Target Vision on Perinatology and Paediatrics 2030^39^ that declares the implementation of telemedicine in usual care as paramount for enhancing the healthcare of rural-dwelling children. In addition, the current state hospital plan of Mecklenburg-Western Pomerania from 2022 defines four paediatric centres that have to provide tele-paediatric services for other hospitals in their catchment areas. Although the telemedicine implementation process and the development of tele-paediatric care centres is still ongoing, this shows the importance of tele-paediatric care concepts for the paediatric healthcare provision in rural regions. Especially, telemedicine centres which organize and plan the tele-consultation requests and services for whole regions could help to overcome the disadvantages of a decentralized tele-paediatric care network which we observed in the RTP-Net project. Those telemedicine centres could also provide technical support and enhanced user training for the hospitals of the network.

#### Research question 3: What improvements to the telemedicine system are needed to enhance users’ technology acceptance and engagement?

The third question in this research sought to determine what improvements of the RTP-Net system are needed. This study revealed that further technical development is needed in order to improve the ease-of-use and, in some cases, the usability of the RTP-Net system. The RTP-Net system would also benefit from integrating digital diagnostic means and remote monitoring tools in order to provide telemedicine for an extended scope of clinical indications.

For instance, this study has been unable to demonstrate the usability of the RTP-Net system in paediatric emergency care even though previous studies highlighted the advantages of telemedicine use for supporting paediatric emergency care departments from rural underserved regions.^30^ Considering emergency care, the RTP-Net system should provide a fast and easy to access video connection between emergency care units of different degrees of specialization in order to discuss the need and urgency of transferrals. According to the participants from paediatric emergency departments, a comprehensive health information exchange is less important than making patients’ vital parameters accessible from remote, for example, by including a multiple camera system that captures and transmits the whole setting in which the patient is situated including the vital parameter monitor screen. This visual information would enable the specialist physician to remotely provide recommendations on resuscitation procedures, artificial ventilation of the patient, other procedures of intensive care as well as on the preparation of a patient transferral.

In contrast to the telemedical emergency care use case, the provision of specialized tele-consultations on certain medical questions would mostly benefit from technical solutions allowing a comprehensive patient health information exchange including previous findings from various examinations (radiology, EEG, sonography, auscultation, etc.). If a second opinion is needed, the transmission of recorded findings would be appropriate to support the clinical decision-making. However, if a physician without a certain specialization is consulting a specialist physician, the specialist physician should have the opportunity to remotely examine the patient health status, for example, by the use of tele-auscultation and tele-EEG, tele-sonography. Previous studies have proven the feasibility and acceptability of these remote examination methods.

Tele-auscultation and tele-EEG support remote diagnostics in paediatric cardiology and neuropaediatrics. It is a promising way to provide comprehensive specialists’ recommendations via telemedicine without the need to transfer a patient to the next specialized hospital. For example, the tele-EEG application can be used for clarifying a suspected epilepsy diagnosis from remote or for monitoring the medication of patient living with epilepsy. The tele-auscultation can be useful for remotely clarifying the need and the urgency for advanced diagnostics, like echocardiography before transferring a patient to a paediatric cardiologist. The advantages of digital diagnostic tools are that they support AI analysis which can help to accurately and efficiently identify abnormalities and that they provide a history of recordings/findings that can be reviewed by different healthcare professionals as needed. Moreover, high accuracy rates were found for tele-otoscopy in patients with otitis media,^40^ tele-echocardiography in neonates,^41^ tele-spirometry in children with asthma^42^ and tele-auscultation in children with pulmonary and heart diseases.^43^ However, it is challenging to integrate these different applications and devices in a comprehensive telemedicine care concept for paediatric patients due to limited interoperability, missing interfaces and a large number of different involved sub-systems used by the participating hospitals, as discussed above.

Overall, this study strengthens the idea that future works could benefit from the development of a standardized framework on the design and implementation of tele-paediatric care networks. The results from this study can contribute to a module on technical aspects within this framework in order to inform future projects about important usability aspects and technical pitfalls to the implementation that need to be addressed.

### Strengths and limitations

To our knowledge, there has been no studies on the implementation and acceptance of comprehensive tele-paediatric care networks with a wide range of paediatric sub-disciplines that seeks to provide telemedical support of cross-organizational patient pathways in a rural underserved region. That is why, the research questions and the design of this study had an explorative/descriptive character and initially tried to expose which regional paediatric pathways exits, which use cases can benefit from using tele-consultations and electronic health information exchange, and which use cases need more advanced telemedical applications, for example, tele-EEG, tele-auscultation, etc. in order to support clinical decision-making adequately. Moreover, the study aimed to explore general implementation barriers and facilitators to the tele-paediatric network. Nonetheless, inferential statistics within a controlled study design could be useful in the future in order to draw correlations between users’ or institutional characteristics and the technology acceptance scores in those hospitals. It also might be useful to assess the implementation of a telemedical care network in another patient group or another healthcare setting in order to evaluate the generalizability of the findings.

The strengths of this study were the integration of both quantitative and qualitative data and methods which, therefore, provides an in-depth understanding of the use cases that can be supported by a tele-paediatric care network. This allowed, also, an identification of the required technical adaptations and further developments needed to extend the scope of the network to other use cases of rural paediatric care. Moreover, implementation facilitators and barriers could be identified from a real-world perspective of the clinical users. The participatory design and the involvement of multiple paediatric departments with different subspecialties strengthened the generalizability, as the results are relevant to other paediatricians. In addition, the iterative implementation process allowed the simultaneous use and adaptation of the RTP-Net system to the local needs of the participating hospitals.

A limitation of the study was that the length of time for administrative processes differed between the participating hospitals, so that some hospitals started later using the RTP-Net system than others. Moreover, the explorative design of the study and the snowball sampling strategy in recruiting specialist physicians caused that some paediatric departments and their corresponding specialties (e.g., neuropaediatrics and paediatric cardiology) were over- or underrepresented. As a consequence, the sample of patients included for prototype testing of the RTP-Net might not be representative. Future studies on the effectiveness of the implementation of telemedicine should consider a randomized and controlled design with a fixed sample size for each telemedical indication and a parallel sampling.

Moreover, frequent staff turnover, especially, of specialist physicians with certain subspecialties temporarily interrupted the provision of specialized tele-consultations in the respective areas. Future implementations of regional tele-paediatric care networks should consider a centralized organization and coordination of the telemedical service delivery in order to compensate interruptions by referring to a surrogate. Moreover, the development of telemedicine centres with a telemedicine care unit responsible for specific catching areas would be necessary.

## Conclusion

The methods used for this study followed an innovative mixed-methods approach that integrated survey data and participant observations and may be applied to other telemedicine implementation projects in complex healthcare settings elsewhere in the world.

This study has clearly shown that a regional tele-paediatric healthcare network can support the inpatient healthcare of children especially in rural regions where specialist physicians are rare and distances to the next paediatrician can be long. The results show that a tele-paediatric care network is able to pool paediatric competences of a large geographical region which makes it a promising approach for low- and mid-income countries that faces similar challenges in providing paediatric healthcare.

However, further technical development and adaptations of the system are needed in order to address further specific uses case of regional paediatric care, such as tele-paediatric emergency care or remote examination and monitoring of patients. Moreover, a nationwide healthcare data infrastructure is needed that supports health information exchange and provides data protection and patient privacy safeguards to foster the users’ trust in the system.

Another important practical implication is that improvements of the reimbursement schedule are needed. First, the reimbursement system should take greater account of financing telemedical services provided by hospitals. Second, the Federal States should fund hospital investments in telemedical equipment and digital diagnostic instruments. In addition, a reform of the G-DRG reimbursement system can foster interfacility cooperation and the use of telemedicine. The introduction of regional budgets is a promising approach since it allows a renumeration per capita rather than per service provided by an individual healthcare provider and, therefore, encourages healthcare providers from a region to find more efficient ways for their collaboration on the patients’ healthcare, for example, by using telemedicine.

Furthermore, the integration of telemedicine training modules into study programs of nursing science and medical science would help to prepare junior staff for future work tasks connected with the evolving field of telemedicine applications. In addition, regular training sessions for healthcare workers at the job could improve the digital and telemedical competences of the healthcare professionals working in the healthcare system.

Finally, the findings reported here shed new light on the relevance of a tele-paediatric care network for the healthcare provision in a resource-limited setting and pointed out the facilitators and barriers to its implementation. Potentially useful adaptations of a tele-paediatric care network and its contribution to the healthcare of rural-dwelling children has been identified and can inform the development of a generic framework on tele-paediatric networks.

## Supplemental Material

sj-doc-1-dhj-10.1177_20552076251350924 - Supplemental material for Implementation of a tele-paediatric network in hospitals in a rural region: A mixed methods implementation study Supplemental material, sj-doc-1-dhj-10.1177_20552076251350924 for Implementation of a tele-paediatric network in hospitals in a rural region: A mixed methods implementation study by Nils Pfeuffer, Angelika Beyer, Luisa Tischler, Sarah Heimbuch, Heiko Krause, Markus Krohn, Steffen Fleßa, Thomas Ruppel, Wolfgang Hoffmann and Neeltje van den Berg in DIGITAL HEALTH

## References

[1] Looi JC Allison S Bastiampillai T , et al. Australian specialised mental healthcare labour shortages: potential interventions for consideration and further research. Australas Psychiatry 2024; 32: 446–449. 39110758 10.1177/10398562241267138 PMC11440790

[2] Liu JX Goryakin Y Maeda A , et al. Global health workforce labor market projections for 2030. Hum Resour Health 2017; 15: 11. 28159017 10.1186/s12960-017-0187-2 PMC5291995

[3] Scheffler RM Campbell J Cometto G , et al. Forecasting imbalances in the global health labor market and devising policy responses. Hum Resour Health 2018; 16: 5. 29325556 10.1186/s12960-017-0264-6 PMC5765602

[4] Holbe F Walus A . Pediatrics in rural regions caught between thrift and ensuring adequate healthcare provision. Gesundheitswesen 2022; 84: 219–226. 33494111 10.1055/a-1327-2614

[5] Beyer A Moon K Penndorf P , et al. Triage through telemedicine in paediatric emergency care—results of a concordance study. PLoS One 2022; 17: e0269058. 10.1371/journal.pone.0269058 PMC9135216 35617339

[6] Vestal E Newman S Phillips S. Barriers and facilitators to accessing pediatric specialty care for rural-dwelling children with complex chronic conditions: an integrative review. J Pediatr Nurs 2024; 77: e385–e393. 10.1016/j.pedn.2024.05.001 38777676

[7] World Health Organization. *Consolidated telemedicine implementation guide*. World Health Organization, 2022. ISBN: 978 92 4 005918 4.

[8] Udeh C Udeh B Rahman N , et al. Telemedicine/virtual ICU: where are we and where are we going? Methodist Debakey Cardiovasc J 2018; 14: 26. 10.14797/mdcj-14-2-126 PMC6027727 29977469

[9] Taylor MA Knochel ML Proctor SJ , et al. Pediatric trauma telemedicine in a rural state: lessons learned from a 1-year experience. J Pediatr Surg 2021; 56: 385–389. 33228973 10.1016/j.jpedsurg.2020.10.020

[10] Hayden EM Boggs KM Espinola JA , et al. Telemedicine facilitation of transfer coordination from emergency departments. Ann Emerg Med 2020; 76: 602–608. 32534835 10.1016/j.annemergmed.2020.04.027 PMC7252127

[11] Haynes SC Dharmar M Hill BC , et al. The impact of telemedicine on transfer rates of newborns at rural community hospitals. Acad Pediatr 2020; 20: 636–641. 32081766 10.1016/j.acap.2020.02.013

[12] Umoren RA Gray MM Schooley N , et al. Effect of video-based telemedicine on transport management of simulated newborns. Air Med J 2018; 37: 317–320. 30322635 10.1016/j.amj.2018.05.007

[13] Alnasser Y Proaño A Loock C , et al. Telemedicine and pediatric care in rural and remote areas of middle-and-low-income countries: narrative review. J Epidemiol Glob Health 2024; 14: 779–786. 38478166 10.1007/s44197-024-00214-8 PMC11442723

[14] Kobel M Kalden P Michaelis A , et al. Accuracy of the apple watch iECG in children with and without congenital heart disease. Pediatr Cardiol 2022; 43: 191–196. 34468775 10.1007/s00246-021-02715-w

[15] Sasangohar F Davis E Kash BA , et al. Remote patient monitoring and telemedicine in neonatal and pediatric settings: scoping literature review. J Med Internet Res 2018; 20: e295. 10.2196/jmir.9403 PMC6320401 30573451

[16] O’Brien DM Dhillon AK Luan-Erfe BM . Impact of telemedicine on patient-centered outcomes in pediatric critical care: a systematic review. Anesthesia Research 2024; 1: 54–66.

[17] Beyer A Moon K Hirsch T , et al. Implementation of a telemedical urgency assessment procedure in the pediatric emergency room: evaluation results. Gesundheitswesen 2024; 86: S275–S281. 2024/05/13. 10.1055/a-2325-0194 38740378

[18] Chen E Leos C Kowitt SD , et al. Enhancing community-based participatory research through human-centered design strategies. Health Promot Pract 2020; 21: 37–48. 31131633 10.1177/1524839919850557

[19] Ryan N Vieira D Gyamfi J , et al. Development of the ASSESS tool: a comprehenSive tool to support rEporting and critical appraiSal of qualitative, quantitative, and mixed methods implementation reSearch outcomes. Implement Sci Commun 2022; 3: 34. 35346390 10.1186/s43058-021-00236-4 PMC8959802

[20] Parker C Scott S Geddes A . Snowball sampling. SAGE research methods foundations. London: Sage Publications Ltd, 2019. DOI: 10.4135/9781526421036831710.

[21] DeJonckheere M Lindquist-Grantz R Toraman S , et al. Intersection of mixed methods and community-based participatory research: a methodological review. J Mix Methods Res 2019; 13: 481–502.

[22] Pfeuffer N Beyer A Penndorf P , et al. Evaluation of a health information exchange system for geriatric health care in rural areas: development and technical acceptance study. JMIR Hum Factors 2022; 9: e34568. 20220915. 10.2196/34568 PMC9523522 36107474

[23] Rivers BM Hernandez ND Rivers D , et al. Utilizing community-based participatory research principles in a safety-net hospital to develop a research partnership. J Health Care Poor Underserved 2019; 30: 27–35. 31735715 10.1353/hpu.2019.0112 PMC7245649

[24] Davis FD . A technology acceptance model for empirically testing new end-user information systems: Theory and results. Cambridge, MA: Massachusetts Institute of Technology, 1985.

[25] Venkatesh V Davis FD. A theoretical extension of the technology acceptance model: four longitudinal field studies. Manage Sci 2000; 46: 186–204.

[26] Garavand A Aslani N Nadri H , et al. Acceptance of telemedicine technology among physicians: a systematic review. Inf Med Unlocked 2022; 30: 100943.

[27] Tan S-H Wong C-K Yap Y-Y , et al. Factors influencing telemedicine adoption among physicians in the Malaysian healthcare system: a revisit. Digit Health 2024; 10: 20552076241257050. 38854922 10.1177/20552076241257050 PMC11159542

[28] Tetik G Türkeli S Pinar S , et al. Health information systems with technology acceptance model approach: a systematic review. Int J Med Inf 2024; 190: 105556. 10.1016/j.ijmedinf.2024.105556 39053345

[29] Seim J . Participant observation, observant participation, and hybrid ethnography. Sociol Methods Res 2024; 53: 121–152.

[30] Mitra A Veerakone R Li K , et al. Telemedicine in paediatric emergency care: a systematic review. J Telemed Telecare 2023; 29: 579–590. 34590883 10.1177/1357633X211010106

[31] Dayal P Chang CH Benko WS , et al. Hospital utilization among rural children served by pediatric neurology telemedicine clinics. JAMA Netw Open 2019; 2: e199364–e199364. 10.1001/jamanetworkopen.2019.9364 PMC6704740 31418803

[32] Fortini S Espeche A Caraballo R . Telemedicine and epilepsy: a patient satisfaction survey of a pediatric remote care program. Epilepsy Res 2020; 165: 106370. 32516743 10.1016/j.eplepsyres.2020.106370

[33] Esposito S Rosafio C Antodaro F , et al. Use of telemedicine healthcare systems in children and adolescents with chronic disease or in transition stages of life: consensus document of the Italian society of telemedicine (SIT), of the Italian society of preventive and social pediatrics (SIPPS), of the Italian society of pediatric primary care (SICuPP), of the Italian federation of pediatric doctors (FIMP) and of the syndicate of family pediatrician doctors (SIMPeF). J Pers Med 2023; 13: 35. 10.3390/jpm13020235 PMC9965862 36836469

[34] Shi J Yan X Wang M , et al. Factors influencing the acceptance of pediatric telemedicine services in China: a cross-sectional study. Front Pediatr. 2021; 9: 745687. 34733810 10.3389/fped.2021.745687 PMC8558490

[35] Bertelsmann Stiftung, Smart Health Systems–Digitalisierung braucht effektive Strategie, politische Führung und eine koordinierende nationale Institution. *Daten, Analysen, Perspektiven* 2018. ISSN: 2364-5970.

[36] Federal Republic of Germany. Gesetz für sichere digitale Kommunikation und Anwendungen im Gesundheitswesen (E-Health-Gesetz). Bundesgesetzblatt 2015. Part I No. 54. Bonn. 29. Decembre 2015.

[37] Federal Republic of Germany. Gesetz für ein Zukunftsprogramm Krankenhäuser (Krankenhauszukunftsgesetz – KHZG). Bundesgesetzblatt 2020. Part I No. 48. Bonn. 28. Octobre 2020.

[38] Federal Republic of Germany. Gesetz zur Beschleunigung der Digitalisierung des Gesundheitswesens (Digital-Gesetz – DigiG). Bundesgesetzblatt 2024. Part I No. 101. Bonn. 25. March 2024.

[39] H ealth Commission of the Federal State of Mecklenburg-Western Pomerania. Zielbild für die Geburtshilfe und Pädiatrie in MV, https://www.regierung-mv.de/Landesregierung/sm/gesundheit/Expertenkommission-zur-Weiterentwicklung-des-Gesundheitswesens/ (2024, accessed 20.03.2025).

[40] Fang T-Y Lin T-Y Shen C-M , et al. Algorithm-driven tele-otoscope for remote care for patients with Otitis Media. Otolaryngol Head Neck Surg 2024; 170: 1590–1597. 38545686 10.1002/ohn.738

[41] Makkar A Milsten J McCoy M , et al. Tele-echocardiography for congenital heart disease screening in a level II neonatal intensive care unit with hybrid telemedicine system. Telemed J E Health 2021; 27: 1136–1142. 33449839 10.1089/tmj.2020.0440

[42] van der Kamp MR Hengeveld VS Brusse-Keizer MGJ , et al. Ehealth technologies for monitoring pediatric asthma at home. scoping review. J Med Internet Res 2023; 25: e45896. 10.2196/45896 PMC10403763 37477966

[43] Fan M Wang Q Liu J , et al. Real-world evaluation of the stemoscope electronic tele-auscultation system. Biomed Eng Online 2022; 21: 63. 36068509 10.1186/s12938-022-01032-4 PMC9446597

