# Supplementary material for: Implementation of a tele-paediatric network in hospitals in a rural region: A mixed methods implementation study
Source: Digit Health. 2025 Jul 20;11:20552076251350924. doi: 10.1177/20552076251350924 (PMC12277559; doi:10.1177/20552076251350924)
Supplement: sj-doc-1-dhj-10.1177_20552076251350924 - Supplemental material for Implementation of a tele-paediatric network in hospitals in a rural region: A mixed methods implementation study [file sj-doc-1-dhj-10.1177_20552076251350924.doc]

# Documentation of the telemedical contacts within the RTP-Net project

**RTP-Net used a web-based user survey with the following items. Responses to the items were collected with help of electronic case report forms on the web-based medical documentation platform (eHealth platform)**

# **Patient master data obtained by creating a new medical case on the eHealth platform**

## Last name, First name

________________________________________________

## Address

________________________________________________

## Gender

☐ male ☐ female ☐ others

## Date of birth

____________________

## Email address

_________________________________________________

## Telephone number

__________________________________________________

|  |
| --- |

# eCRF: Admission Form (to be filled out by the telemedicine requesting physician for each tele-consultation)

## My location (name and place of the clinic).

____________________________________________

## Anamnesis.

____________________________________________________________________________________________________________________________________________________________________________________________________________________________________________________________________________________________________________________

## Main diagnosis at admission (ICD-10-code).

__________________________________________________________________________________________________________________________________________________________

## Secondary diagnoses at admission (ICD-10-code).

__________________________________________________________________________________________________________________________________________________________

## Diagnosis for which the tele-consultation was requested (ICD-10-code).

________________________________________________________________________________________________________________________________________________________

## Question for the tele-consultation.

________________________________________________________________________________________________________________________________________________________

## Consequences of the tele-consultation:

| ☐ | Referring patient to the outpatient emergency service | ☐ | Referring patient to the responsible family doctors practice during the office hours | | ☐ | Hospitalisation at the tele-consultation requesting  hospital |
| --- | --- | --- | --- | --- | --- | --- |
| ☐ | Transfer of the patient to a hospital of the next higher healthcare level | ☐ | Change of the ongoing treatment | | ☐ | Nothing |
| ☐ | Others: _______________________________ | | |  | | |

# eCRF: Tele-consultation report (to be filled out by the telemedicine providing physician for each tele-consultation)

## Start time of the consultation

Date: ____________ time: _____________

## My location (name and place of the hospital)

________________________________________________

## Anamnesis.

____________________________________________________________________________________________________________________________________________________________________________________________________________________________________________________________________________________________________________________

## Please indicate your medical specialization:

|  | | ☐ | Paediatric oncology and haematology | | | ☐ | Paediatric cardiology | | | ☐ | Neonatology | |
| --- | --- | --- | --- | --- | --- | --- | --- | --- | --- | --- | --- | --- |
|  | | ☐ | Neuropediatric | | | ☐ | Paediatric Endocrinology | | | ☐ | Paediatric diabetology | |
|  | | ☐ | Paediatric gastroenterology | | | ☐ | Paediatric nephrology | | | ☐ | Paediatric pneumology | |
|  | | ☐ | Paediatric rheumatology | | | ☐ | Paediatric orthopaedics | | | ☐ | Paediatric Allergology | |
| ☐ | Others:  ___________________ | | |  |  | | |  |  | | |  |

## Recommendations to the tele-consultation requesting physician (considering therapy, medication, further diagnostics, prognosis)

___________________________________________________________________________________________________________________________________________________________________________________________________________________________________________________________________________________________________________________________________________________________________________________________________________________________________________________________________________________________________________________________________________________________

## Treatment diagnosis after tele-consultation (ICD-10-code)

__________________________________________________________________________________________________________________________________________________________

## End time of the consultation

Date: ____________ Time: _____________

# eCRF: Evaluation of the user satisfaction and technology acceptance (to be filled out by both sides after each telemedical contact)

## I was satisfied with the course of the tele-consultation.

☐ strongly agree ☐ agree ☐ neutral

☐ disagree ☐ totally disagree ☐ do not know

## Did technical issues occur during the course of the tele-consultation?

☐ yes ☐ no

## If so, which technical issues occurred?

|  |  | | |  |  | | |  |  | | |  |
| --- | --- | --- | --- | --- | --- | --- | --- | --- | --- | --- | --- | --- |
|  | | ☐ | Connection failed | | | ☐ | Disconnection during the tele-consultation | | | ☐ | Insufficient audio quality | |
|  | | ☐ | Insufficient video quality | | | ☐ | Others:  __________________________ | | |  |  | |

## Did organizational issues occur during the course of the tele-consultation?

☐ yes ☐ no

## If so, which organizational issues occurred?

__________________________________________________________________________________________________________________________________

## Was the tele-consultation adequate for usual paediatric care?

☐ yes ☐ no

## If not, why was the tele-consultation for this case inadequate?

___________________________________________________________________________________________________________________________________________________________________________________________________

_____________________________________________________________________________
